# Effects of Spironolactone on Arrhythmias in Hemodialysis Patients: Secondary Results of the SPin-D Randomized Controlled Trial

**DOI:** 10.34067/KID.0000000000000067

**Published:** 2023-02-10

**Authors:** Finnian R. Mc Causland, Jesse Y. Hsu, Jonathan Himmelfarb, Talat Alp Ikizler, Dominic S. Raj, Rajnish Mehrotra, Sushrut S. Waikar, Paul L. Kimmel, Alan S. Kliger, Laura M. Dember, David M. Charytan

**Affiliations:** 1 Renal Division, Department of Medicine, Brigham and Women's Hospital, Boston, Massachusetts; 2 Harvard Medical School, Boston, Massachusetts; 3 Department of Biostatistics, Epidemiology and Informatics, Perelman School of Medicine, University of Pennsylvania, Philadelphia, Pennsylvania; 4 Center for Clinical Epidemiology and Biostatistics, Perelman School of Medicine, University of Pennsylvania, Philadelphia, Pennsylvania; 5 Kidney Research Institute, Division of Nephrology, Department of Medicine, University of Washington, Seattle, Washington; 6 Division of Nephrology and Hypertension, Department of Medicine, and Vanderbilt Center for Kidney Disease, Vanderbilt University Medical Center, Nashville, Tennessee; 7 Division of Renal Diseases and Hypertension, George Washington University School of Medicine, Washington, DC; 8 Section of Nephrology, Department of Medicine, Boston University School of Medicine and Boston Medical Center, Boston, Massachusetts; 9 National Institute of Diabetes Digestive and Kidney Diseases, Bethesda, Maryland; 10 Department of Medicine, Yale School of Medicine, New Haven, Connecticut; 11 Renal, Electrolyte and Hypertension Division, Perelman School of Medicine, University of Pennsylvania, Philadelphia, Pennsylvania; 12 Nephrology Division, Department of Medicine, New York University Grossman School of Medicine, New York, New York; 13 NYU Langone Health, New York, New York

**Keywords:** randomized trial, spironolactone, ESRD, arrhythmia, wearable electrocardiography

## Abstract

**Key Points:**

- The effects of spironolactone on arrhythmia in patients receiving maintenance hemodialysis are unclear.
- In these *post hoc* analyses, spironolactone resulted in a higher frequency of bradycardia and conduction blocks, compared with placebo.
- Close monitoring may be warranted for patients on maintenance hemodialysis receiving MRAs, while definitive trial results are awaited.

**Background:**

Patients receiving maintenance hemodialysis (HD) have a high incidence of cardiovascular events, including arrhythmia and sudden death. Spironolactone reduces the risk of cardiovascular events and sudden death in patients with heart failure, but the effects of spironolactone on arrhythmic events in patients treated with maintenance HD are unclear.

**Methods:**

The Safety and Cardiovascular Efficacy of Spironolactone in Dialysis-Dependent ESRD (SPin-D) trial was a 36-week randomized, placebo-controlled, double-blind trial comparing three different doses of spironolactone with placebo in maintenance HD patients. We performed a *post hoc* analysis in a subset (*n*=57) who underwent extended electrocardiographic monitoring using a wearable device at baseline and follow-up. Generalized estimating equations models were fit to determine the associations of spironolactone (individual doses and combined) versus placebo on the incidence rate of predefined categories of arrhythmic events.

**Results:**

The average age of participants was 55±12 years, 61% were male, and 77% were Black. The overall proportion of patients with at least one arrhythmia event was 43% (15/35) at baseline and 81% (43/53) at the end of follow-up. At the end of follow-up, the rate of bradycardic events or conduction blocks was higher in the combined spironolactone group, compared with placebo (82.4 versus 38.7 events/100 patient-days; *P*<0.001). Similar findings were noted in adjusted models, but did not meet statistical significance (adjusted rate ratio of 2.04; 95% confidence interval 0.83–5.05).

**Conclusions:**

In a 36-week trial of patients receiving maintenance HD, a higher frequency of bradycardia and conduction blocks was observed among those treated with spironolactone treatment compared with placebo. Larger studies are required to investigate the longer-term effects of spironolactone on cardiac conduction in patients receiving HD.

## Graphical abstract

**Figure:**
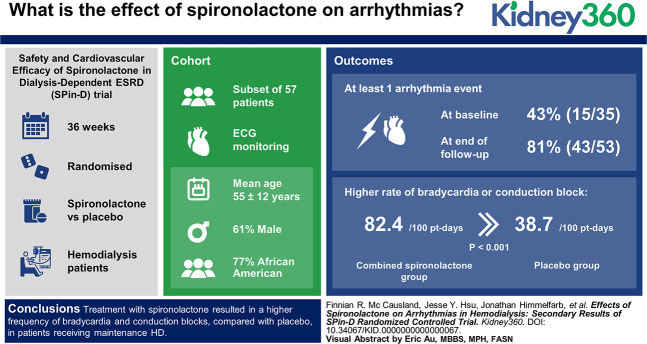

## Introduction

Cardiovascular disease accounts for most of the deaths in patients receiving maintenance hemodialysis (HD).^1^ Approximately half of cardiovascular mortality events are attributed to sudden cardiac death,^1^ and adverse cardiac events (including dysrhythmia) occur with greater frequency on days during which HD is performed.^2–5^ The development of arrhythmia is hypothesized to be a major risk factor for sudden cardiac death, and the frequency of arrhythmic events also seems to be temporally related to the HD schedule.^6^

While inhibitors of the renin-angiotensin-aldosterone system have been shown to reduce morbidity and mortality in non-HD patients with a variety of cardiac pathologies,^7–10^ evidence supporting the use of renin-angiotensin-aldosterone system inhibition in patients requiring maintenance HD is less clear.^11,12^ Aldosterone is known to be a key mediator of adverse myocardial remodeling and fibrosis,^13^ processes that may predispose to arrhythmias. As aldosterone concentrations are often elevated in HD patients, there is a biologic rationale for pharmacologic blockade of the mineralocorticoid receptor. However, enthusiasm for using mineralocorticoid receptor antagonists in patients receiving HD is tempered by concerns about hyperkalemia.^14,15^

The Safety and Cardiovascular Efficacy of Spironolactone in Dialysis-Dependent ESRD (SPin-D) study was a double-blind randomized clinical trial designed to assess safety and tolerability of three doses of spironolactone versus placebo in patients receiving maintenance HD (*n*=129).^16^ Overall, spironolactone was well tolerated, with no major difference in the frequency of hyperkalemia >6.5 mEq/L or serious hypotension compared with placebo but had no detectable effect on measures of diastolic function in exploratory efficacy analyses. A subset of 57 SPin-D participants underwent continuous electrocardiographic monitoring for 7-day periods at baseline, 6 weeks, and/or between weeks 32–36, using a wearable device. *Post hoc* analysis of electrocardiographic data allowed us to test the hypothesis that spironolactone reduces the risk of arrhythmic events, compared with placebo.

## Methods

SPin-D was a parallel-group, double-blind, randomized, multiple-dosage trial designed to test the safety and tolerability of spironolactone versus placebo in adult patients receiving maintenance HD (NCT02285920). Participants were enrolled from 13 HD units affiliated with four academic medical centers in the United States and were randomized in a 2:1:1:1 ratio to placebo or spironolactone 12.5, 25, or 50 mg daily for 36 weeks. The institutional review boards affiliated with each site approved the protocol, and each participant provided written informed consent.

### Study Population and Procedures

SPin-D enrolled 129 participants who met the following inclusion criteria: age 18–85 years and treatment with maintenance HD for >6 months or for 3–6 months if there were no changes in target dry weight during the prior 2 weeks and no hospitalizations during the previous 6 weeks. Notable exclusion criteria included serum potassium concentration ≥6.5 mEq/L or unscheduled dialysis for hyperkalemia within 3 months; serum potassium concentration ≥6.0 mEq/L within 2 weeks before baseline; pre-HD systolic blood pressure <100 mm Hg within 2 weeks before screening or at baseline; ≥2 dialysis sessions within the month before screening with blood pressure <80 mm Hg or treatment for cramping, light-headedness, nausea, or hypotension; use of digoxin, spironolactone, or eplerenone; or dual use of angiotensin-converting enzyme inhibitors and angiotensin receptor blockers.

A protocol amendment was added on January 22, 2016, after 64 patients had enrolled in the trial to include optional cardiac monitoring during trial participation. A subset of 57 patients consented to undergo seven days of continuous heart rate and rhythm monitoring using a wearable patch (SEEQ Mobile Cardiac Telemetry System; Medtronic, Minneapolis, MN) before randomization, at week 6, and during weeks 32–36. Thirty (53%) of the 57 participants completed electrocardiographic monitoring at all three time points.

### Exposures and Outcomes

The primary exposure for these *post hoc* subgroup analyses was the randomized treatment arm, with comparisons of the individual dosage arms of spironolactone with placebo. The secondary exposure of interest was spironolactone, regardless of dose, evaluated by combining the three spironolactone groups for comparisons with placebo.

The Medtronic SEEQ devices were preprogrammed to detect 26 arrhythmia events. Because some of the arrhythmia events were rare, the outcomes for the present analyses considered six aggregated categories of arrhythmia events as follows: (1) atrial fibrillation/flutter on ≥41 of 45 consecutive beats at ≥150 beats per minute; (2) ventricular arrhythmia defined as polymorphic or monomorphic ventricular tachycardia on ≥18 of 20 beats; (3) conduction blocks defined as asystole >3 seconds, first degree atrioventricular block, second degree atrioventricular block, or junctional rhythm or intraventricular conduction delay; (4) bradycardia defined as <40 beats per minute on four of five consecutive beats; (5) atrial/sinus or other nonventricular tachycardias; and (6) conduction blocks or bradycardia (Supplemental Table 1).

### Study Data

Baseline data for trial participants were obtained through self-report, medical record review, and questionnaires. In addition to blood for batched analyses collected at baseline and at 36 weeks, pre-HD serum potassium concentration was measured at the following time points: every month; 3–5 days and 2 weeks after each study drug dose increase; and within 1 week after any hyperkalemia events (defined as any serum potassium concentration >6.0 mEq/L, whether obtained for the trial or for clinical purposes). Echocardiography was performed at baseline and at the end of follow-up.

### Statistical Analyses

Continuous variables were examined graphically and recorded as mean (± SD) for normally distributed data or median (with 25th and 75th percentile) for non-normally distributed data. Categorical variables were examined by frequency distribution and recorded as proportions. Differences across randomized treatment arms were assessed using analysis of variance, chi-squared test, or Kruskal-Wallis tests, as appropriate.

Crude recurrent arrhythmia events were presented as frequency (%) and incidence rate (per 100 patient-days) by treatment groups at baseline, week 6, and the end of study. *P*-values for linear trends using equally spaced scores, and for comparisons between the combined spironolactone groups and placebo group, were determined using generalized estimating equations with independent correlation structure accounting for clustering effect of centers and participants: specifically, Poisson distribution with a log link for recurrent arrythmia events. Models included the exposure of interest (*i.e.*, treatment groups), discrete study visits, trial randomization stratification factors of time since initiating dialysis (<1 or ≥1 year), and current angiotensin converting enzyme inhibitor or angiotensin receptor blocker use (yes/no). Rate ratios (RRs) for recurrent arrhythmia events were estimated from the generalized estimating equations models for the composite of atrial fibrillation or flutter and the composite of bradycardia or conduction blocks. Estimated RRs for other arrhythmia events were not generated because of insufficient numbers of events. Exploratory analyses were also performed to determine the association of various predictors with the composite outcomes of atrial fibrillation/flutter and bradycardia or conduction block. To avoid overfitting, the number of covariates included in the adjusted models was based, in part, on the frequency of outcome events.

All analyses were performed using SAS version 9.4 (SAS Institute Inc.) and geepack package in R version 3.4.3 (https://www.r-project.org). Given the pilot nature of the current analyses, with a focus on exploration, no corrections were made for multiple comparisons.

## Results

### Baseline Characteristics

The baseline characteristics of the SPin-D participants who did (*n*=57) and did not (*n*=72) take part in the electrocardiographic monitoring substudy are presented in Supplemental Table 2. Of those included in this analysis, most of the participants were Black (77%), 61% were male, the mean age was 55±12 years, and the median duration on HD was 3.4 (25th–75th percentile 1.9–6.9) years. The baseline characteristics were similar among the four randomized treatment arms (Table 1) and in the combined spironolactone and placebo groups (Supplemental Table 3). The baseline echocardiographic parameters were generally similar across randomized treatment arms, with the exception of left ventricular (LV) end-diastolic diameter and body surface area–adjusted LV mass, which were both lower in the 50 mg spironolactone group (Supplemental Table 4).

**Table 1:** Baseline characteristics

Characteristic	All (*n*=57)	Placebo (*n*=21)	Spironolactone 12.5 mg (*n*=11)	Spironolactone 25 mg (*n*=12)	Spironolactone 50 mg (*n*=13)
Male, *n* (%)	35 (61.4)	13 (61.9)	4 (36.4)	8 (66.7)	10 (76.9)
Age, yr; mean (SD)	55.3 (12.1)	53.8 (10.8)	52.7 (16.8)	58.8 (13.3)	56.6 (7.9)
Black, *n* (%)	44 (77.2)	18 (85.7)	8 (72.7)	9 (75.0)	9 (69.2)
White, *n* (%)	7 (12.3)	0 (0.00)	3 (27.3)	2 (16.7)	2 (15.4)
Asian, *n* (%)	3 (5.3)	1 (4.8)	0 (0.00)	1 (8.3)	1 (7.7)
Hispanic/Latino, *n* (%)	5 (8.8)	2 (9.5)	2 (18.2)	0 (0.00)	1 (7.7)
BMI, kg/m^2^; mean (SD)	32.6 (7.9)	30.4 (6.5)	33.6 (10.7)	33.1 (8.7)	34.7 (6.5)
Systolic BP, mm Hg; mean (SD)	138.7 (22.2)	140.6 (24.1)	134.5 (16.9)	138.9 (30.0)	139.2 (15.6)
Diastolic BP, mm Hg; mean (SD)	75.1 (10.1)	74.7 (8.2)	72.5 (8.9)	73.7 (9.5)	79.2 (13.9)
Hypertension, *n* (%)	55 (96.5)	21 (100.0)	11 (100.0)	11 (91.7)	12 (92.3)
Diabetes mellitus, *n* (%)	36 (63.2)	12 (57.1)	7 (63.6)	7 (58.3)	10 (76.9)
Coronary artery disease, *n* (%)	18 (31.6)	6 (28.6)	1 (9.1)	5 (41.7)	6 (46.2)
Congestive heart failure, *n* (%)	10 (17.5)	3 (14.3)	2 (18.2)	3 (25.0)	2 (15.4)
Atrial fibrillation, *n* (%)	5 (8.8)	3 (14.3)	0 (0.00)	2 (16.7)	0 (0.00)
Stroke, *n* (%)	10 (17.5)	6 (28.6)	2 (18.2)	0 (0.00)	2 (15.4)
Peripheral vascular disease, *n* (%)	11 (19.3)	3 (14.3)	2 (18.2)	4 (33.3)	2 (15.4)
Hyperlipidemia, *n* (%)	28 (49.1)	9 (42.9)	6 (54.5)	6 (50.0)	7 (53.8)
Current tobacco use, *n* (%)	8 (14.0)	3 (14.3)	1 (9.1)	1 (8.3)	3 (23.1)
AV graft, *n* (%)	7 (12.3)	2 (9.5)	4 (36.4)	0 (0.00)	1 (7.7)
AV fistula, *n* (%)	48 (84.2)	19 (90.5)	7 (63.6)	11 (91.7)	11 (84.6)
Tunneled CVC, *n* (%)	1 (1.8)	0 (0.00)	0 (0.00)	0 (0.00)	1 (7.7)
Other, *n* (%)	1 (1.8)	0 (0.00)	0 (0.00)	1 (8.3)	0 (0.00)
Dialysis vintage, yr; median 25th-75th percentile	3.4 (1.9–6.9)	3.9 (2.2–10.4)	1.5 (1.0–3.7)	3.6 (2.8–5.5)	3.4 (2.2–4.9)
Dialysis ≥1 year, *n* (%)	52 (91.2)	20 (95.2)	8 (72.7)	12 (100.0)	12 (92.3)
ACEI or ARB use, *n* (%)	18 (31.6)	7 (33.3)	3 (27.3)	3 (25.0)	5 (38.5)
Beta blockers use, *n* (%)	31 (54.4)	11 (52.4)	6 (54.5)	6 (50.0)	8 (61.5)
Statins use, *n* (%)	30 (52.6)	9 (42.9)	7 (63.6)	7 (58.3)	7 (53.8)
Antiplatelet agents use, *n* (%)	25 (43.9)	8 (38.1)	5 (45.5)	6 (50.0)	6 (46.2)
Single pool Kt/V; mean (SD)	1.6 (0.4)	1.4 (0.2)	1.5 (0.2)	1.6 (0.4)	1.8 (0.6)
24-h urine volume, ml; mean (SD)^a^	202.5 (299.8)	205.7 (345.1)	182.1 (233.2)	255.7 (372.0)	169.9 (226.0)

Sample sizes in the header represent numbers of patients with at least one monitoring done at baseline, week 6, or the end of study. There were no statistically significant differences between groups at baseline. BMI, body mass index; AV, arteriovenous; CVC, central venous catheter; ACEi, angiotensin-converting enzyme inhibitor; ARB, angiotensin receptor blocker.

a Values determined for 55 of the 57 participants.

### Arrhythmia Events at Baseline, 6 Weeks, and End of Follow-Up, According to Randomized Treatment Arm

**Figure 1:**
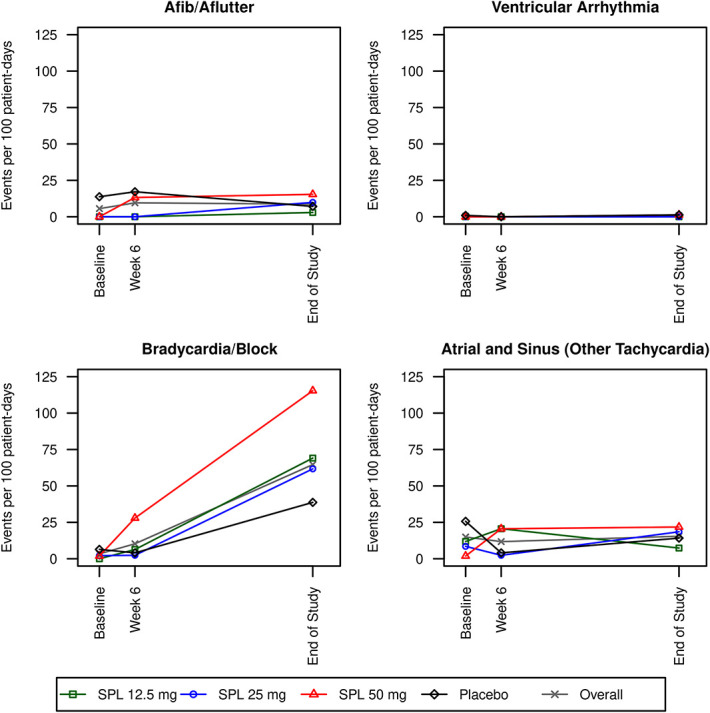
**Arrhythmia event rates by treatment groups and study visits.** SPL, spironolactone.

Among the 35 SPin-D participants who underwent electrocardiographic monitoring at baseline, nine of 14 (64%) in the placebo group and six of 21 (29%) in the combined spironolactone group had at least one arrhythmia event during the baseline monitoring period (Figure 1 and Table 2). Most of these events were atrial/sinus and other nonventricular tachycardias, followed in frequency by atrial fibrillation/flutter, and conduction blocks (frequency of individual events are provided in Supplemental Tables 5-9). Overall arrhythmia event rates at baseline were lower for the individual and combined spironolactone groups, compared with placebo (Supplemental Table 10).

**Table 2:** Crude arrhythmia events

Arrhythmia Type		Placebo (*n*=21)		Spironolactone Groups Combined (*n*=36)		Spironolactone 12.5 mg (*n*=11)		Spironolactone 25 mg (*n*=12)		Spironolactone 50 mg (*n*=13)	
		Pts w/Event *n* (%)	Events per 100 Pt-Days	Pts w/Event *n* (%)	Events per 100 Pt-Days	Pts w/Event *n* (%)	Events per 100 Pt-Days	Pts w/Event *n* (%)	Events per 100 Pt-Days	Pts w/Event *n* (%)	Events per 100 Pt-Days
Baseline	Monitoring done, *n*	14		21		8		6		7	
	Atrial fibrillation/flutter	2 (14.3)	13.8	0 (0.0)	0	0 (0.0)	0	0 (0.0)	0	0 (0.0)	0
	Ventricular arrhythmia	1 (7.1)	0.9	0 (0.0)	0	0 (0.0)	0	0 (0.0)	0	0 (0.0)	0
	Conduction block	3 (21.4)	5.5	1 (4.8)	0.6	0 (0.0)	0	1 (16.7)	2.1	0 (0.0)	0
	Bradycardia	1 (7.1)	0.9	1 (4.8)	0.6	0 (0.0)	0	0 (0.0)	0	1 (14.3)	1.9
	Atrial/sinus/other nonventricular tachycardia	4 (28.6)	25.7	4 (19.0)	7.5	2 (25.0)	11.9	1 (16.7)	8.5	1 (14.3)	1.9
	Bradycardia or conduction block	4 (28.6)	6.4	2 (9.5)	1.3	0 (0.0)	0	1 (16.7)	2.1	1 (14.3)	1.9
	Any arrhythmia events	9 (64.3)	53.2	6 (28.6)	10.1	2 (25.0)	11.9	2 (33.3)	12.8	2 (28.6)	5.7
Week 6	Monitoring done, *n*	14		23		8		6		9	
	Atrial fibrillation/flutter	2 (15.4)	17.2	3 (13.0)	5.2	0 (0.0)	0	0 (0.0)	0	3 (33.3)	13.2
	Ventricular arrhythmia	0 (0.0)	0	0 (0.0)	0	0 (0.0)	0	0 (0.0)	0	0 (0.0)	0
	Conduction block	1 (7.7)	1.0	5 (21.7)	7.5	2 (25.0)	3.2	1 (16.7)	2.4	2 (22.2)	14.7
	Bradycardia	1 (7.7)	3.0	4 (17.4)	6.4	2 (25.0)	3.2	0 (0.0)	0	2 (22.2)	13.2
	Atrial/sinus/other nonventricular tachycardia	2 (15.4)	4.0	5 (21.7)	16.2	2 (25.0)	20.6	1 (16.7)	2.4	2 (22.2)	20.6
	Bradycardia or conduction block	2 (15.4)	4.0	7 (30.4)	13.9	3 (37.5)	6.3	1 (16.7)	2.4	3 (33.3)	27.9
	Any arrhythmia events	4 (30.8)	29.3	11 (47.8)	49.1	3 (37.5)	33.3	2 (33.3)	7.1	6 (66.7)	89.7
End of study	Monitoring done, *n*	21		32		9		11		12	
	Atrial fibrillation/flutter	4 (19.0)	7.1	4 (12.5)	9.7	1 (11.1)	2.9	1 (9.1)	9.9	2 (16.7)	15.4
	Ventricular arrhythmia	1 (4.8)	1.3	1 (3.1)	0.4	0 (0.0)	0	0 (0.0)	0	1 (8.3)	1.3
	Conduction block	13 (61.9)	38.7	20 (62.5)	76.2	7 (77.8)	69.1	6 (54.5)	61.7	7 (58.3)	97.4
	Bradycardia	0 (0.0)	0	1 (3.1)	6.2	0 (0.0)	0	0 (0.0)	0	1 (8.3)	17.9
	Atrial/sinus/other nonventricular tachycardia	8 (38.1)	14.2	11 (34.4)	16.3	3 (33.3)	7.4	4 (36.4)	18.5	4 (33.3)	21.8
	Bradycardia or conduction block	13 (61.9)	38.7	20 (62.5)	82.4	7 (77.8)	69.1	6 (54.5)	61.7	7 (58.3)	115.4
	Any arrhythmia events	18 (85.7)	100.0	25 (78.1)	191.2	7 (77.8)	148.5	9 (81.8)	151.9	9 (75.0)	269.2

Sample sizes in the header represent numbers of patients with at least one monitoring done at baseline, week 6, or the end of study. Pts, participants.

A total of 37 patients underwent electrocardiographic monitoring at 6 weeks. The proportion of patients with any event was 31% (4 of 14 patients) in the placebo group and 48% (11 of 23 patients) in the combined spironolactone group (Figure 1 and Table 2). There were no major differences in event rates based on the randomized treatment groups at the 6-week time point (Supplemental Table 10).

A total of 53 patients underwent electrocardiographic monitoring at the end of follow-up. The proportion of patients with any event was 86% (18 of 21 patients) in the placebo group and 78% (25 of 32 patients) in the combined spironolactone group (Figure 1 and Table 2). The combined spironolactone group had a higher rate of bradycardia or conduction blocks, compared with the placebo group (82.4 events per 100 patient-days versus 38.7 events per 100 patient-days; *P*<0.001 [Supplemental Table 10]).

Ventricular events were rare during the baseline, 6-week, and end of follow-up monitoring periods.

### Effect of Spironolactone Versus Placebo on Atrial Fibrillation/Flutter and Bradycardia or Conduction Blocks

The higher frequency of atrial fibrillation/flutter and bradycardia or conduction block events, compared with other events, permitted the estimation of RRs, according to the randomized groups. There were no significant associations of spironolactone (individual dose groups or combined) with atrial fibrillation/flutter, compared with placebo (Table 3).

**Table 3:** Estimated rate ratios (95% confidence intervals) for atrial fibrillation/flutter and bradycardia/conduction blocks, according to randomized treatment group

Model	Exposure	Atrial Fibrillation or Atrial Flutter^a^	Bradycardia or Conduction Blocks^b^
1	Spironolactone 12.5 mg vs placebo	0.09 (0.01–1.04)	1.56 (0.73–3.34)
	Spironolactone 25 mg vs placebo	0.40 (0.03–5.01)	1.45 (0.61–3.44)
	Spironolactone 50 mg vs placebo	0.89 (0.11–6.96)	3.00 (0.80–11.20)
2	Spironolactone combined vs placebo	0.47 (0.07–3.21)	2.04 (0.83–5.05)

a Models (independence correlation) included the exposure of interest and study visits.

b Models (independence correlation) included the exposure of interest, study visits, length of time on dialysis, and current angiotensin converting enzyme inhibitor/angiotensin receptor blocker use.

A dose-related, but nonsignificant, increase in bradycardia or conduction block events with spironolactone was observed, with a RR of 3.0 (95% confidence interval [CI] 0.80–11.20) for the 50 mg group and 1.45 (95% CI 0.61–3.44) for the 25 mg group, compared with placebo. Similarly, there was an approximately two-fold higher risk of bradycardia or conduction block events for the combined spironolactone group versus placebo (RR 2.04; 95% CI 0.83–5.05 [Table 3]). In a sensitivity analysis, examining only patients with available baseline and follow-up data (*n*=33), the combined spironolactone group again had a nominally higher, but nonsignificant, risk of bradycardia or conduction block events, compared with placebo (RR 1.80 [95% CI, 0.82 to 3.97]).

### Exploratory Analyses for the Association of Other Variables of Interest with Atrial Fibrillation/Flutter and Bradycardia or Conduction Blocks

Exploratory analyses were performed to evaluate associations of variables of interest including hyperkalemia, intradialytic hypotension, ultrafiltration volume, and echocardiographic measures, with risks of atrial fibrillation/flutter and bradycardia or conduction blocks. Higher baseline left atrial diameter was associated with an increased risk of atrial fibrillation/flutter (RR, 3.02; 95% CI, 1.13 to 8.07), but not with bradycardia or conduction block (RR, 1.64; 95% CI, 0.62 to 4.34). Although hyperkalemia within the prior 7 days (versus none) had the highest effect estimate for risk of bradycardia or conduction block events, this did not reach statistical significance (RR, 2.07; 95% CI, 0.52 to 8.25). Effect estimates for the association of hyperkalemia (defined in several ways) with atrial fibrillation/flutter were also nonsignificant [Supplemental Table 11]).

## Discussion

In this *post hoc* analysis of cardiac rhythm monitoring performed in a subset of participants in the SPin-D trial, we found that spironolactone resulted in a higher frequency of bradycardic or conduction block events compared with placebo and that this risk was most pronounced at the highest evaluated dose of 50 mg/d. However, adjusted effect estimates, while directionally consistent with the crude observations, did not meet statistical significance.

Although one of the major effects of mineralocorticoid receptor stimulation by aldosterone is to promote renal sodium reabsorption and potassium secretion in the distal nephron, the mineralocorticoid receptor is known to be present in many other cell types, with overactivation being associated with development of fibrosis of the heart,^17^ vasculature,^18^ and kidney.^19,20^ In animal models, blockade of the mineralocorticoid receptor has been shown to ameliorate LV hypertrophy and myocardial fibrosis,^21-24^ providing additional support for a potential pathogenic role of excessive aldosterone concentrations. Cardiac structural abnormalities are common in patients initiating HD^25^ and are often characterized by myocardial fibrosis and reduced capillary density, compared with normal and hypertensive controls.^26^ This constellation of findings may predispose to the development of myocardial hypoxia and conduction system dysfunction, generating conditions for the development of arrhythmia.^27^ In light of these associations, and the presence of elevated aldosterone concentrations in patients receiving maintenance HD,^28,29^ there has been much interest in the use of mineralocorticoid receptor antagonists as a potential therapeutic option to reduce adverse cardiovascular outcomes in this high-risk patient population.

Atrial fibrillation is the most common clinically evident arrhythmia in HD patients and has been found to occur in up to 40% of patients in studies using implantable loop recorders.^6,30,31^ In the non-HD population, the evidence supporting the use of mineralocorticoid receptor antagonists to prevent atrial fibrillation is somewhat conflicting. While several systematic reviews and meta-analyses of randomized controlled trials and observational studies have suggested a beneficial association of such agents for the reduction of new-onset and recurrent atrial fibrillation,^32,33^ in contrast, a *post hoc* analysis of the TOPCAT (Treatment of Preserved Cardiac Function Heart Failure with an Aldosterone Antagonist) trial did not find evidence for beneficial effects of spironolactone on reduction of incident atrial fibrillation in patients with heart failure and preserved ejection fraction.^34^ In this respect, it is notable that the mean left ventricular ejection fraction in this study was 67.5%. Trials testing the ability of mineralocorticoid receptor antagonists to reduce arrhythmic risk in patients with ESKD are lacking, but observational data have suggested a decreased risk of new-onset atrial fibrillation among those prescribed, compared with those not prescribed, spironolactone.^35^ In this study, which was limited by baseline between-group differences in event rates and low overall event rates, an effect of spironolactone on atrial fibrillation was not detected.

Recent studies using implantable loop recorders in patients receiving maintenance HD have consistently reported a relatively high frequency of bradycardic and conduction block events.^6,36–39^ In the present analyses, we observed a two-fold higher risk of bradycardic and conduction block events with spironolactone use, particularly for the 50 mg dose, compared with placebo at the end of the study, despite a baseline event rate that was higher in the placebo group. Previous studies have reported associations between higher pre-HD serum potassium concentrations and greater risk of arrhythmia-related hospitalizations or sudden death.^40^ Furthermore, data from some implantable loop recorder studies have suggested a higher risk of conduction block/bradycardia with hyperkalemia,^38^ suggesting a potential pathophysiological mechanism that may underlie these observations. While the primary analysis of SPin-D reported no major difference in the frequency of the two primary safety endpoints (hyperkalemia >6.5 mEq/L and serious hypotension) between the combined spironolactone group and placebo, there were trends toward higher frequency of hyperkalemic events with the highest (50 mg) spironolactone dose.^16^ Similarly, the Mineralocorticoid Receptor Antagonists in End-Stage Renal Disease trial (*n*=97), which tested 50 mg spironolactone against placebo over the 40-week follow-up, reported that moderate hyperkalemia (6.0–6.5 mEq/L) was more common in the spironolactone group, but severe hyperkalemia (>6.5 mEq/L) was not.^15^ Similar trends toward higher risks of hyperkalemia were noted in a trial of eplerenone, compared with placebo.^14^ Although nonsignificant and hypothesis-generating, the present findings suggest close monitoring of serum potassium concentration in patients treated with maintenance HD who are prescribed mineralocorticoid receptor antagonists is reasonable while more definitive data are awaited.

There are notable strengths to our present analyses, including the use of continuous electrocardiographic monitoring, the evaluation of multiple doses of spironolactone with placebo comparisons, and the recruitment and detailed follow-up of patients from multiple centers in the setting of a randomized controlled trial. However, there are several limitations to be acknowledged. Only a subset of SPin-D participants were included in the continuous monitoring component of the trial, follow-up was relatively short, and patients were younger and had better preservation of ejection fraction than the overall HD population in the United States, thus limiting generalizability. Not all patients in the monitoring substudy contributed arrhythmia data at all time points, raising the possibility of some element of selection bias and precluding analyses according to the interdialytic interval. Furthermore, the small sample size and lack of blood collection that was standardized with respect to the timing of the rhythm monitoring periods limited the ability to conduct explanatory and mechanistic analyses. Finally, owing to the modest sample size, the categories of prespecified arrhythmias were necessarily broad with small numbers of events precluding meaningful subgroup analyses—thus, the relative clinical importance of subtypes within a category may also not be comparable.

In summary, in this *post hoc* analysis of SPin-D, those who received spironolactone had a higher risk of bradycardia or conduction blocks, as assessed by continuous electrocardiography, compared with those who received placebo. However, while hypothesis-generating, the present analyses were underpowered and should be interpreted as such while we await the results of definitive trials examining hard clinical endpoints (NCT01848639 and NCT03020303).

## Supplementary Material

SUPPLEMENTARY MATERIAL

## Data Availability

Anonymized data created for the study are or will be available in a persistent repository on publication: Analyzable Data, NIDDK Repository.

## Supplemental Material

Supplemental Table 1. Preprogrammed arrhythmia events detected by the Medtronic SEEQ device and *a priori* aggregated arrhythmia events.

Supplemental Table 2. Characteristics of the SPin-D trial participants at baseline according to participation or nonparticipation in the electrocardiographic monitoring substudy.

Supplemental Table 3. Characteristics of the patients at baseline, according to the combined spironolactone arms or placebo.

Supplemental Table 4. Characteristics of echocardiography at baseline, according to the randomized treatment assignment.

Supplemental Table 5. Aggregated and individual conduction block events.

Supplemental Table 6. Aggregated and individual atrial fibrillation/flutter events.

Supplemental Table 7. Aggregated and individual ventricular arrhythmia events.

Supplemental Table 8. Aggregated and individual bradycardia events.

Supplemental Table 9. Aggregated and individual atrial/sinus/other nonventricular tachycardia events.

Supplemental Table 10. Arrhythmia event rates (per 100 patient-days) and 95% confidence intervals.

Supplemental Table 11. Estimated rate ratios (95% CIs) of other exposures of interest for atrial fibrillation/flutter and bradycardia/conduction blocks.
