# Supplementary material for: Effects of Spironolactone on Arrhythmias in Hemodialysis Patients: Secondary Results of the SPin-D Randomized Controlled Trial
Source: Kidney360. 2023 Feb 10;4(4):e486–95. doi: 10.34067/KID.0000000000000067 (PMC10278797; doi:10.34067/KID.0000000000000067)
Supplement: SUPPLEMENTARY MATERIAL [file kidney360-4-e486-s001.pdf]

Supplementary Table 1. Preprogrammed arrhythmia events detected by the Medtronic SEEQ device and a priori aggregated arrhythmia events

Preprogrammed Event	Aggregated Event for Analyses
Atrial Fibrillation	Atrial Fibrillation/Flutter
Atrial Flutter	
Monomorphic VT	Ventricular Arrhythmia
Polymorphic VT/VF	
1st Degree AVB	Conduction Block
2nd Degree AVB(2:1)	
2nd Degree AVB-(Mobitz I)	
2nd Degree AVB-(Mobitz II)	
IVCD	
Junctional Rhythm	
Pause	Bradycardia
Sinus Bradycardia	
Sinus Tachycardia	Atrial/Sinus/Other Non-Ventricular Tachycardias
Supraventricular Tachycardia	
Wide Complex Tachycardia	
3rd Degree AVB	Low events rates in the remaining categories precluded further analysis.
Artifact	
High Degree AVB	
ICD Discharge	
Idioventricular rhythm	
Other	
Paced	
PACs/PJCs	
PVCs	
Sinus Arrhythmia	
Sinus Rhythm	

Abbreviations: VT, ventricular tachycardia; VF, ventricular fibrillation; AVB, atrio-ventricular block; IVCD, inter-ventricular conduction delay; ICD, Implantable cardioverter-defibrillator; PAC, premature atrial complexes; PJC, premature junctional complexes; PVC, premature ventricular complexes

Supplementary Table 2. Characteristics of the SPin-D Trial Participants at Baseline According to Participation or Non-Participation in the Electrocardiographic Monitoring Sub-Study

	**Participants (n = 57)**	**Non-Participants (n = 72)**
Male, n (%)	35 (61.4%)	50 (69.4%)
Age, yr; mean (SD)	55.3 (12.1)	55.7 (12.1)
Black, n (%)	44 (77.2%)	48 (66.7%)
White, n (%)	7 (12.3%)	20 (27.8%)
Asian, n (%)	3 (5.3%)	2 (2.8%)
Hispanic/Latino, n (%)	5 (8.8%)	6 (8.3%)
BMI, kg/m^2; mean (SD)	32.6 (7.9)	30.6 (6.8)
Systolic BP, mm Hg; mean (SD)	138.7 (22.2)	140.4 (22.3)
Diastolic BP, mm Hg; mean (SD)	75.1 (10.1)	79.0 (13.3)
Hypertension, n (%)	55 (96.5%)	65 (90.3%)
Diabetes mellitus, n (%)	36 (63.2%)	30 (41.7%)
Coronary artery disease, n (%)	18 (31.6%)	10 (13.9%)
Congestive heart failure, n (%)	10 (17.5%)	11 (15.3%)
Atrial fibrillation, n (%)	5 (8.8%)	5 (6.9%)
Stroke, n (%)	10 (17.5%)	12 (16.7%)
Peripheral vascular disease, n (%)	11 (19.3%)	5 (6.9%)
Hyperlipidemia, n (%)	28 (49.1%)	21 (29.2%)
Current tobacco use, n (%)	8 (14.0%)	10 (13.9%)
AV graft, n (%)	7 (12.3%)	7 (9.7%)
AV fistula, n (%)	48 (84.2%)	61 (84.7%)
Tunneled CVC, n (%)	1 (1.8%)	1 (1.4%)
Other, n (%)	1 (1.8%)	3 (4.2%)
Dialysis vintage, yr; median (25th-75th percentile)	3.4 (1.9 - 6.9)	3.6 (2.0 - 5.9)
Dialysis ≥1 year, n (%)	52 (91.2%)	64 (88.9%)
ACEI or ARB use, n (%)	18 (31.6%)	21 (29.2%)
Beta blockers use, n (%)	31 (54.4%)	30 (41.7%)
Statins use, n (%)	30 (52.6%)	24 (33.3%)
Anti-platelet agents use, n (%)	25 (43.9%)	19 (26.4%)
Single pool Kt/V; mean (SD)	1.6 (0.4)	1.5 (0.2)
24-hr urine volume, ml; mean (SD)	202.5 (299.8)	139.4 (261.9)

Abbreviations: BMI, body mass index; BP, blood pressure; SD, standard deviation; AV, arteriovenous; CVC, central venous catheter; ACEi, angiotensin converting enzyme inhibitor; ARB, angiotensin receptor blocker

Supplementary Table 3. Characteristics of the Patients at Baseline, According to the Combined Spironolactone Arms or Placebo

	All (n=57)	Placebo (n=21)	Spironolactone Combined (n=36)
Male, n (%)	35 (61.4)	13 (61.9)	22 (61.1)
Age, yr; mean (SD)	55.3 (12.1)	53.8 (10.8)	56.2 (12.8)
Black, n (%)	44 (77.2)	18 (85.7)	26 (72.2)
White, n (%)	7 (12.3)	0 (0.00)	7 (19.4)
Asian, n (%)	3 (5.3)	1 (4.8)	2 (5.6)
Hispanic/Latino, n (%)	5 (8.8)	2 (9.5)	3 (8.3)
BMI; mean (SD)	32.6 (7.9)	30.4 (6.5)	33.8 (8.4)
Systolic BP, mm Hg; mean (SD)	138.7 (22.2)	140.6 (24.1)	137.7 (21.3)
Diastolic BP, mm Hg; mean (SD)	75.1 (10.1)	74.7 (8.2)	75.3 (11.2)
Hypertension, n (%)	55 (96.5)	21 (100.0)	34 (94.4)
Diabetes mellitus, n (%)	36 (63.2)	12 (57.1)	24 (66.7)
Coronary artery disease, n (%)	18 (31.6)	6 (28.6)	12 (33.3)
Congestive heart failure, n (%)	10 (17.5)	3 (14.3)	7 (19.4)
Atrial fibrillation, n (%)	5 (8.8)	3 (14.3)	2 (5.6)
Stroke, n (%)	10 (17.5)	6 (28.6)	4 (11.1)
Peripheral vascular disease, n (%)	11 (19.3)	3 (14.3)	8 (22.2)
Hyperlipidemia, n (%)	28 (49.1)	9 (42.9)	19 (52.8)
Current tobacco use, n (%)	8 (14.0)	3 (14.3)	5 (13.9)
AV graft, n (%)	7 (12.3)	2 (9.5)	5 (13.9)
AV fistula, n (%)	48 (84.2)	19 (90.5)	29 (80.6)
Tunneled CVC, n (%)	1 (1.8)	0 (0.00)	1 (2.8)
Other, n (%)	1 (1.8)	0 (0.00)	1 (2.8)
Dialysis vintage, yr; median (IQR)	3.4 (1.9 - 6.9)	3.9 (2.2 - 10.4)	3.4 (1.5 - 4.9)
Dialysis ≥1year, n (%)	52 (91.2)	20 (95.2)	32 (88.9)
ACEI or ARB use, n (%)	18 (31.6)	7 (33.3)	11 (30.6)
Beta blockers use, n (%)	31 (54.4)	11 (52.4)	20 (55.6)
Statins use, n (%)	30 (52.6)	9 (42.9)	21 (58.3)
Anti-platelet agents use, n (%)	25 (43.9)	8 (38.1)	17 (47.2)
Single pool Kt/V; mean (SD)	1.6 (0.4)	1.4 (0.2)	1.6 (0.4)
24-hr urine volume, ml; mean (SD)[a]	202.5 (299.8)	205.7 (345.1)	200.7 (276.0)

Abbreviations: BMI, body mass index; BP, blood pressure; SD, standard deviation; AV, arteriovenous; CVC, central venous catheter; ACEi, angiotensin converting enzyme inhibitor; ARB, angiotensin receptor blocker

*Sample sizes in the heading represent numbers of patients with at least one monitoring performed at baseline,
Week 6, or the end of study.

[a]Values determined for 55 of the 57 participants.

Supplementary Table 4. Characteristics of Echocardiography at Baseline, According to the Randomized Treatment Assignment

	All (n=57)	Placebo (n=21)	Spironolactone 12.5 mg (n=11)	Spironolactone 25 mg (n=12)	Spironolactone 50 mg (n=13)
E/E' lateral[a]	11.6 (6.3)	11.9 (7.6)	10.2 (5.7)	10.4 (5.7)	13.1 (5.3)
LV mass adjusted for BSA[b], g/m^2	106.0 (24.6)	100.8 (26.9)	105.1 (20.1)	123.3 (29.2)	99.1 (9.8)
LV ejection fraction 2D[b], %	67.5 (8.5)	69.4 (3.5)	67.2 (9.4)	64.1 (13.7)	67.9 (7.4)
LV end-diastolic diameter, mm	4.6 (0.6)	4.6 (0.4)	4.9 (0.8)	4.6 (0.4)	4.3 (0.4)
LV end-systolic diameter, mm	2.9 (0.6)	2.9 (0.4)	3.2 (1.0)	2.9 (0.6)	2.8 (0.4)
LV end-diastolic volume adjusted for BSA[b], mL/m^2	60.5 (14.6)	57.9 (11.9)	70.0 (20.8)	56.3 (4.3)	58.7 (13.9)
LV end-systolic volume adjusted for BSA[b], mL/m^2	20.4 (11.6)	19.6 (9.5)	27.2 (21.1)	18.2 (5.0)	18.1 (5.6)
LV stroke volume adjusted for BSA[b], mL/m^2	40.1 (8.0)	38.3 (7.1)	42.8 (9.4)	38.1 (4.1)	40.6 (9.1)
LV cardiac output adjusted for BSA[b], mL/min/m^2	3162.1 (681.0)	2948.3 (732.1)	3423.9 (674.5)	3142.8 (535.4)	3135.5 (729.6)
LV fractional shortening, %	36.8 (7.4)	36.3 (7.5)	36.1 (9.1)	38.1 (9.2)	36.6 (5.3)
LV global long strain[c], %	-17.0 (3.3)	-17.5 (2.4)	-16.4 (3.9)	-17.1 (4.4)	-16.5 (2.9)
LA diameter, mm	3.8 (0.4)	3.8 (0.4)	3.6 (0.5)	3.9 (0.4)	4.0 (0.3)
LA volume adjusted for BSA[b], mL/m^2	34.2 (11.7)	30.8 (6.9)	34.5 (9.4)	33.7 (9.3)	36.2 (15.6)

Abbreviations: E/E'; ratio between early mitral inflow velocity and mitral annular early diastolic velocity; LV, left ventricular; BSA, body surface area; 2D, two dimensional; LA, left atrial

[a]Values determined for 51 of the 57 participants.

[b]Values determined for 56 of the 57 participants.

[c]Values determined for 53 of the 57 participants.

*Sample sizes in the heading represent numbers of patients with at least one monitoring performed at baseline, Week 6, or the end of study.

There were significant differences between groups for LV mass adjusted for BSA (P=0.04) and LV end-diastolic diameter (P=0.04)

Supplementary Table 5. Aggregated and individual conduction block events

		Placebo (n=21)		Spironolactone Groups Combined (n=36)		Spironolactone 12.5 mg (n=11)		Spironolactone 25 mg (n=12)		Spironolactone 50 mg (n=13)	
		Pts w/event n (%)	Events per 100 pt-days	Pts w/event n (%)	Events per 100 pt-days	Pts w/event n (%)	Events per 100 pt-days	Pts w/event n (%)	Events per 100 pt-days	Pts w/event n (%)	Events per 100 pt-days
Baseline	Monitoring Done, n	14		21		8		6		7	
	1st Degree AVB	3 (21.4)	2.8	0 (0.0)	0.0	0 (0.0)	0.0	0 (0.0)	0.0	0 (0.0)	0.0
	2nd Degree AVB(2:1)	0 (0.0)	0.0	0 (0.0)	0.0	0 (0.0)	0.0	0 (0.0)	0.0	0 (0.0)	0.0
	2nd Degree AVB-(Mobitz I)	0 (0.0)	0.0	0 (0.0)	0.0	0 (0.0)	0.0	0 (0.0)	0.0	0 (0.0)	0.0
	2nd Degree AVB-(Mobitz II)	0 (0.0)	0.0	0 (0.0)	0.0	0 (0.0)	0.0	0 (0.0)	0.0	0 (0.0)	0.0
	IVCD	2 (14.3)	1.8	1 (4.8)	0.6	0 (0.0)	0.0	1 (16.7)	2.1	0 (0.0)	0.0
	Junctional Rhythm	1 (7.1)	0.9	0 (0.0)	0.0	0 (0.0)	0.0	0 (0.0)	0.0	0 (0.0)	0.0
	Conduction Block	3 (21.4)	5.5	1 (4.8)	0.6	0 (0.0)	0	1 (16.7)	2.1	0 (0.0)	0
Week 6	Monitoring Done, n	14		23		8		6		9	
	1st Degree AVB	3 (21.4)	5.5	1 (4.8)	0.6	0 (0.0)	0.0	1 (16.7)	2.1	0 (0.0)	0.0
	2nd Degree AVB(2:1)	0 (0.0)	0.0	1 (4.3)	0.6	0 (0.0)	0.0	1 (16.7)	2.4	0 (0.0)	0.0
	2nd Degree AVB-(Mobitz I)	0 (0.0)	0.0	1 (4.3)	1.2	0 (0.0)	0.0	0 (0.0)	0.0	1 (11.1)	2.9
	2nd Degree AVB-(Mobitz II)	0 (0.0)	0.0	2 (8.7)	1.7	1 (12.5)	1.6	0 (0.0)	0.0	1 (11.1)	2.9
	IVCD	0 (0.0)	0.0	1 (4.3)	1.7	0 (0.0)	0.0	0 (0.0)	0.0	1 (11.1)	4.4
	Junctional Rhythm	1 (7.7)	1.0	2 (8.7)	1.7	1 (12.5)	1.6	0 (0.0)	0.0	1 (11.1)	2.9
	Conduction Block	1 (7.7)	1.0	5 (21.7)	7.5	2 (25.0)	3.2	1 (16.7)	2.4	2 (22.2)	14.7
End of Study	Monitoring Done, n	21		32		9		11		12	
	1st Degree AVB	5 (23.8)	12.3	5 (15.6)	6.6	2 (22.2)	4.4	1 (9.1)	7.4	2 (16.7)	7.7
	2nd Degree AVB(2:1)	0 (0.0)	0.0	1 (3.1)	2.2	0 (0.0)	0.0	0 (0.0)	0.0	1 (8.3)	6.4
	2nd Degree AVB-(Mobitz I)	0 (0.0)	0.0	1 (3.1)	4.8	0 (0.0)	0.0	0 (0.0)	0.0	1 (8.3)	14.1
	2nd Degree AVB-(Mobitz II)	0 (0.0)	0.0	0 (0.0)	0.0	0 (0.0)	0.0	0 (0.0)	0.0	0 (0.0)	0.0
	IVCD	11 (52.4)	26.5	19 (59.4)	62.1	6 (66.7)	64.7	6 (54.5)	54.3	7 (58.3)	67.9
	Junctional Rhythm	0 (0.0)	0.0	1 (3.1)	0.4	0 (0.0)	0.0	0 (0.0)	0.0	1 (8.3)	1.3
	Conduction Block	13 (61.9)	38.7	20 (62.5)	76.2	7 (77.8)	69.1	6 (54.5)	61.7	7 (58.3)	97.4

Abbreviations: VT, ventricular tachycardia; VF, ventricular fibrillation;
AVB, atrio-ventricular block; IVCD, inter-ventricular conduction delay; ICD, Implantable cardioverter-defibrillator;
PAC, premature atrial complexes; PJC, premature junctional complexes; PVC, premature ventricular complexes

*Sample sizes in the heading represent numbers of patients with at least one monitoring performed at baseline, Week 6, or the end of study.

Supplementary Table 6. Aggregated and individual atrial fibrillation/flutter events

		Placebo (n=21)		Spironolactone Groups Combined (n=36)		Spironolactone 12.5 mg (n=11)		Spironolactone 25 mg (n=12)		Spironolactone 50 mg (n=13)	
		Pts w/event n (%)	Events per 100 pt-days	Pts w/event n (%)	Events per 100 pt-days	Pts w/event n (%)	Events per 100 pt-days	Pts w/event n (%)	Events per 100 pt-days	Pts w/event n (%)	Events per 100 pt-days
Baseline	Monitoring Done, n	14		21		8		6		7	
	Atrial Fibrillation	2 (14.3)	12.8	0 (0.0)	0.0	0 (0.0)	0.0	0 (0.0)	0.0	0 (0.0)	0.0
	Atrial Flutter	1 (7.1)	0.9	0 (0.0)	0.0	0 (0.0)	0.0	0 (0.0)	0.0	0 (0.0)	0.0
	Atrial Fibrillation/Flutter	2 (14.3)	13.8	0 (0.0)	0	0 (0.0)	0	0 (0.0)	0	0 (0.0)	0
Week 6	Monitoring Done, n	14		23		8		6		9	
	Atrial Fibrillation	2 (15.4)	15.2	3 (13.0)	4.6	0 (0.0)	0.0	0 (0.0)	0.0	3 (33.3)	11.8
	Atrial Flutter	1 (7.7)	2.0	1 (4.3)	0.6	0 (0.0)	0.0	0 (0.0)	0.0	1 (11.1)	1.5
	Atrial Fibrillation/Flutter	2 (15.4)	17.2	3 (13.0)	5.2	0 (0.0)	0	0 (0.0)	0	3 (33.3)	13.2
End of Study	Monitoring Done, n	21		32		9		11		12	
	Atrial Fibrillation	3 (14.3)	6.5	4 (12.5)	9.7	1 (11.1)	2.9	1 (9.1)	9.9	2 (16.7)	15.4
	Atrial Flutter	1 (4.8)	0.6	0 (0.0)	0.0	0 (0.0)	0.0	0 (0.0)	0.0	0 (0.0)	0.0
	Atrial Fibrillation/Flutter	4 (19.0)	7.1	4 (12.5)	9.7	1 (11.1)	2.9	1 (9.1)	9.9	2 (16.7)	15.4

*Sample sizes in the heading represent numbers of patients with at least one monitoring performed at baseline, Week 6, or the end of study.

Supplementary Table 7. Aggregated and individual ventricular arrhythmia events

		Placebo (n=21)		Spironolactone Groups Combined (n=36)		Spironolactone 12.5 mg (n=11)		Spironolactone 25 mg (n=12)		Spironolactone 50 mg (n=13)	
		Pts w/event n (%)	Events per 100 pt-days	Pts w/event n (%)	Events per 100 pt-days	Pts w/event n (%)	Events per 100 pt-days	Pts w/event n (%)	Events per 100 pt-days	Pts w/event n (%)	Events per 100 pt-days
Baseline	Monitoring Done, n	14		21		8		6		7	
	Monomorphic VT	1 (7.1)	0.9	0 (0.0)	0.0	0 (0.0)	0.0	0 (0.0)	0.0	0 (0.0)	0.0
	Polymorphic VT/VF	0 (0.0)	0.0	0 (0.0)	0.0	0 (0.0)	0.0	0 (0.0)	0.0	0 (0.0)	0.0
	Ventricular Arrhythmia	1 (7.1)	0.9	0 (0.0)	0	0 (0.0)	0	0 (0.0)	0	0 (0.0)	0
Week 6	Monitoring Done, n	14		23		8		6		9	
	Monomorphic VT	0 (0.0)	0.0	0 (0.0)	0.0	0 (0.0)	0.0	0 (0.0)	0.0	0 (0.0)	0.0
	Polymorphic VT/VF	0 (0.0)	0.0	0 (0.0)	0.0	0 (0.0)	0.0	0 (0.0)	0.0	0 (0.0)	0.0
	Ventricular Arrhythmia	0 (0.0)	0	0 (0.0)	0	0 (0.0)	0	0 (0.0)	0	0 (0.0)	0
End of Study	Monitoring Done, n	21		32		9		11		12	
	Monomorphic VT	0 (0.0)	0.0	1 (3.1)	0.4	0 (0.0)	0.0	0 (0.0)	0.0	1 (8.3)	1.3
	Polymorphic VT/VF	1 (4.8)	1.3	0 (0.0)	0.0	0 (0.0)	0.0	0 (0.0)	0.0	0 (0.0)	0.0
	Ventricular Arrhythmia	1 (4.8)	1.3	1 (3.1)	0.4	0 (0.0)	0	0 (0.0)	0	1 (8.3)	1.3

Abbreviations: VT, ventricular tachycardia; VF, ventricular fibrillation

*Sample sizes in the heading represent numbers of patients with at least one monitoring performed at baseline, Week 6, or the end of study.

Supplementary Table 8. Aggregated and individual bradycardia events

		Placebo (n=21)		Spironolactone Groups Combined (n=36)		Spironolactone 12.5 mg (n=11)		Spironolactone 25 mg (n=12)		Spironolactone 50 mg (n=13)	
		Pts w/event n (%)	Events per 100 pt-days	Pts w/event n (%)	Events per 100 pt-days	Pts w/event n (%)	Events per 100 pt-days	Pts w/event n (%)	Events per 100 pt-days	Pts w/event n (%)	Events per 100 pt-days
Baseline	Monitoring Done, n	14		21		8		6		7	
	Pause	0 (0.0)	0.0	0 (0.0)	0.0	0 (0.0)	0.0	0 (0.0)	0.0	0 (0.0)	0.0
	Sinus Bradycardia	1 (7.1)	0.9	1 (4.8)	0.6	0 (0.0)	0.0	0 (0.0)	0.0	1 (14.3)	1.9
	Bradycardia	1 (7.1)	0.9	1 (4.8)	0.6	0 (0.0)	0	0 (0.0)	0	1 (14.3)	1.9
Week 6	Monitoring Done, n	14		23		8		6		9	
	Pause	0 (0.0)	0.0	1 (4.3)	2.9	0 (0.0)	0.0	0 (0.0)	0.0	1 (11.1)	7.4
	Sinus Bradycardia	1 (7.7)	3.0	4 (17.4)	3.5	2 (25.0)	3.2	0 (0.0)	0.0	2 (22.2)	5.9
	Bradycardia	1 (7.7)	3.0	4 (17.4)	6.4	2 (25.0)	3.2	0 (0.0)	0	2 (22.2)	13.2
End of Study	Monitoring Done, n	21		32		9		11		12	
	Pause	0 (0.0)	0.0	1 (3.1)	6.2	0 (0.0)	0.0	0 (0.0)	0.0	1 (8.3)	17.9
	Sinus Bradycardia	0 (0.0)	0.0	0 (0.0)	0.0	0 (0.0)	0.0	0 (0.0)	0.0	0 (0.0)	0.0
	Bradycardia	0 (0.0)	0	1 (3.1)	6.2	0 (0.0)	0	0 (0.0)	0	1 (8.3)	17.9

*Sample sizes in the heading represent numbers of patients with at least one monitoring performed at baseline, Week 6, or the end of study.

Supplementary Table 9. Aggregated and individual atrial/sinus/other non-ventricular tachycardia events

		Placebo (n=21)		Spironolactone Groups Combined (n=36)		Spironolactone 12.5 mg (n=11)		Spironolactone 25 mg (n=12)		Spironolactone 50 mg (n=13)	
		Pts w/event n (%)	Events per 100 pt-days	Pts w/event n (%)	Events per 100 pt-days	Pts w/event n (%)	Events per 100 pt-days	Pts w/event n (%)	Events per 100 pt-days	Pts w/event n (%)	Events per 100 pt-days
Baseline	Monitoring Done, n	14		21		8		6		7	
	Sinus Tachycardia	4 (28.6)	24.8	3 (14.3)	6.9	2 (25.0)	11.9	1 (16.7)	8.5	0 (0.0)	0.0
	Supraventricular Tachycardia	1 (7.1)	0.9	1 (4.8)	0.6	0 (0.0)	0.0	0 (0.0)	0.0	1 (14.3)	1.9
	Wide Complex Tachycardia	0 (0.0)	0.0	0 (0.0)	0.0	0 (0.0)	0.0	0 (0.0)	0.0	0 (0.0)	0.0
	Atrial/Sinus/Other Non-Ventricular Tachycardia	4 (28.6)	25.7	4 (19.0)	7.5	2 (25.0)	11.9	1 (16.7)	8.5	1 (14.3)	1.9
Week 6	Monitoring Done, n	14		23		8		6		9	
	Sinus Tachycardia	1 (7.7)	1.0	2 (8.7)	6.9	1 (12.5)	12.7	0 (0.0)	0.0	1 (11.1)	5.9
	Supraventricular Tachycardia	1 (7.7)	3.0	2 (8.7)	8.1	1 (12.5)	7.9	0 (0.0)	0.0	1 (11.1)	13.2
	Wide Complex Tachycardia	0 (0.0)	0.0	2 (8.7)	1.2	0 (0.0)	0.0	1 (16.7)	2.4	1 (11.1)	1.5
	Atrial/Sinus/Other Non-Ventricular Tachycardia	2 (15.4)	4.0	5 (21.7)	16.2	2 (25.0)	20.6	1 (16.7)	2.4	2 (22.2)	20.6
End of Study	Monitoring Done, n	21		32		9		11		12	
	Sinus Tachycardia	8 (38.1)	13.5	10 (31.2)	15.4	3 (33.3)	7.4	3 (27.3)	16.0	4 (33.3)	21.8
	Supraventricular Tachycardia	1 (4.8)	0.6	1 (3.1)	0.9	0 (0.0)	0.0	1 (9.1)	2.5	0 (0.0)	0.0
	Wide Complex Tachycardia	0 (0.0)	0.0	0 (0.0)	0.0	0 (0.0)	0.0	0 (0.0)	0.0	0 (0.0)	0.0
	Atrial/Sinus/Other Non-Ventricular Tachycardia	8 (38.1)	14.2	11 (34.4)	16.3	3 (33.3)	7.4	4 (36.4)	18.5	4 (33.3)	21.8

*Sample sizes in the heading represent numbers of patients with at least one monitoring performed at baseline, Week 6, or the end of study.

Supplementary Table 10. Arrhythmia Event Rates (per 100 patient-days) and 95% Confidence Intervals

		Placebo (n=21)	Spironolactone Groups Combined (n=36)	Spironolactone 12.5 mg (n=11)	Spironolactone 25 mg (n=12)	Spironolactone 50 mg (n=13)	P values for Event Rates*	
							Trend	Combined SPL groups vs Placebo
Baseline	Monitoring Done, n	14	21	8	6	7		
	Atrial Fibrillation/Flutter	13.8 (2.9 - 66.2)	0.0 (0.0 - 0.0)	0.0 (0.0 - 0.0)	0.0 (0.0 - 0.0)	0.0 (0.0 - 0.0)	<0.001	<0.001
	Ventricular Arrhythmia	0.9 (0.1 - 5.6)	0.0 (0.0 - 0.0)	0.0 (0.0 - 0.0)	0.0 (0.0 - 0.0)	0.0 (0.0 - 0.0)	0.9	0.4
	Conduction Block	5.5 (2.9 - 10.3)	0.6 (0.1 - 4.0)	0.0 (0.0 - 0.0)	2.1 (0.4 - 10.8)	0.0 (0.0 - 0.0)	0.1	<0.001
	Bradycardia	0.9 (0.1 - 6.0)	0.6 (0.1 - 4.0)	0.0 (0.0 - 0.0)	0.0 (0.0 - 0.0)	1.9 (0.4 - 9.9)	0.6	0.8
	Atrial/Sinus/Other Non-Ventricular Tachycardia	25.7 (6.9 - 95.1)	7.5 (4.5 - 12.7)	11.9 (5.0 - 28.3)	8.5 (1.7 - 43.2)	1.9 (0.4 - 9.9)	0.1	<0.001
	Bradycardia or Conduction Block	6.4 (4.0 - 10.3)	1.3 (0.5 - 3.0)	0.0 (0.0 - 0.0)	2.1 (0.4 - 10.8)	1.9 (0.4 - 9.9)	0.2	0.03
	Any Arrhythmia Events	53.2 (37.2 - 76.0)	10.1 (7.4 - 13.7)	11.9 (5.0 - 28.3)	12.8 (6.0 - 27.1)	5.7 (2.6 - 12.4)	<0.001	<0.001
Week 6	Monitoring Done, n	14	23	8	6	9		
	Atrial Fibrillation/Flutter	16.2 (4.7 - 55.6)	5.2 (1.7 - 16.3)	0.0 (0.0 - 0.0)	0.0 (0.0 - 0.0)	13.2 (4.9 - 35.8)	<0.001	0.2
	Ventricular Arrhythmia	0.0 (0.0 - 0.0)	0.0 (0.0 - 0.0)	0.0 (0.0 - 0.0)	0.0 (0.0 - 0.0)	0.0 (0.0 - 0.0)	NA	NA
	Conduction Block	1.0 (0.2 - 5.8)	7.5 (3.6 - 15.7)	3.2 (1.5 - 6.6)	2.4 (0.5 - 11.4)	14.7 (4.8 - 45.4)	0.1	0.1
	Bradycardia	2.9 (0.5 - 17.3)	6.4 (2.4 - 17.2)	3.2 (1.5 - 6.8)	0.0 (0.0 - 0.0)	13.2 (3.5 - 50.2)	0.01	0.6
	Atrial/Sinus/Other Non-Ventricular Tachycardia	3.8 (1.3 - 11.1)	16.2 (9.2 - 28.4)	20.6 (9.1 - 46.8)	2.4 (0.5 - 11.4)	20.6 (4.8 - 88.7)	0.5	0.1
	Bradycardia or Conduction Block	3.8 (1.3 - 11.1)	13.9 (6.1 - 31.6)	6.3 (3.9 - 10.4)	2.4 (0.5 - 11.4)	27.9 (8.5 - 92.3)	0.1	0.3
	Any Arrhythmia Events	27.6 (16.2 - 47.2)	49.1 (28.3 - 85.3)	33.3 (20.0 - 55.5)	7.1 (3.6 - 14.3)	89.7 (35.6 - 225.9)	0.6	0.5
End of Study	Monitoring Done, n	21	32	9	11	12		
	Atrial Fibrillation/Flutter	7.1 (2.6 - 19.3)	9.7 (5.5 - 17.1)	2.9 (0.5 - 16.4)	9.9 (1.3 - 73.1)	15.4 (6.2 - 38.0)	0.3	0.7
	Ventricular Arrhythmia	1.3 (0.2 - 8.2)	0.4 (0.1 - 3.0)	0.0 (0.0 - 0.0)	0.0 (0.0 - 0.0)	1.3 (0.2 - 7.6)	0.9	0.4
	Conduction Block	38.7 (30.9 - 48.5)	76.2 (68.3 - 85.1)	69.1 (63.3 - 75.5)	61.7 (52.0 - 73.3)	97.4 (62.3 - 152.4)	<0.001	<0.001
	Bradycardia	0.0 (0.0 - 0.0)	6.2 (1.1 - 33.5)	0.0 (0.0 - 0.0)	0.0 (0.0 - 0.0)	17.9 (2.1 - 153.1)	<0.001	0.001
	Atrial/Sinus/Other Non-Ventricular Tachycardia	14.2 (11.1 - 18.1)	16.3 (13.4 - 19.8)	7.4 (3.9 - 13.7)	18.5 (11.7 - 29.2)	21.8 (13.6 - 34.9)	0.2	0.6
	Bradycardia or Conduction Block	38.7 (30.9 - 48.5)	82.4 (70.4 - 96.4)	69.1 (63.3 - 75.5)	61.7 (52.0 - 73.3)	115.4 (64.7 - 205.8)	<0.001	<0.001

	Any Arrhythmia Events	100.0 (88.1 - 113.5)	191.2 (166.6 - 219.5)	148.5 (135.5 - 162.8)	151.9 (136.5 - 168.9)	269.2 (162.4 - 446.4)	<0.001	<0.001

* P-values were calculated using generalized estimating equations with a Poisson distribution with a log link and an independent correlation structure accounting for clustering effect of centers and participants

*Sample sizes in the heading represent numbers of patients with at least one monitoring performed at baseline, Week 6, or the end of study.

Supplementary Table 11. Estimated rate ratios (95% CIs) of other exposures of interest for atrial fibrillation/flutter and bradycardia/conduction blocks.

Model	Exposure of Interest*	Atrial Fibrillation/ Flutter	Bradycardia/ Conduction Blocks
1	Hyperkalemia (within 7 days) vs none	0.20 (0.03 - 1.50)[a]	2.07 (0.52 - 8.25)[b]
2	Hyperkalemia (last 4 observations) vs none	0.98 (0.35 - 2.77)[a]	1.50 (0.44 - 5.06)[b]
3	Hyperkalemia (ever) vs none	0.86 (0.27 - 2.73)[a]	1.52 (0.73 - 3.14)[b]
4	Intra-dialytic hypotension (within 48 hours) vs none	NA[c]	0.47 (0.08 - 2.68)[d]
5	Intra-dialytic fluid removal (last intra-dialytic weight decrease), Kg	0.63 (0.45 - 0.89)[e]	0.94 (0.70 - 1.26)[d]
6	Mean potassium (within 7 days), mEq/L	1.54 (0.89 - 2.65)[f]	1.20 (0.78 - 1.86)[b]
7	Mean potassium (last 4 observations), mEq/L	0.94 (0.28 - 3.18)[a]	1.09 (0.64 - 1.83)[b]
8	Baseline E/E’ lateral ratio	1.02 (0.97 - 1.07)[e]	1.03 (0.99 - 1.07)[d]
9	Baseline LV mass adjusting for BSA, g/m^2	1.02 (0.99 - 1.04)[e]	1.01 (0.98 - 1.03)[d]
10	Baseline LV ejection fraction 2D, %	1.02 (0.93 - 1.12)[e]	0.99 (0.97 - 1.01)[d]
11	Baseline LV global longitudinal strain, %	1.20 (0.94 - 1.54)[e]	0.98 (0.91 - 1.06)[d]
12	Baseline LA diameter, mm	3.02 (1.13 - 8.07)[e]	1.64 (0.62 - 4.34)[d]

Abbreviations: E/E’; ratio between early mitral inflow velocity and mitral annular early diastolic velocity; LV, left ventricular; 2D, two dimensional; LA, left atrial

*Sample sizes for the models differ based on the exposure of interest due to varied missingness

[a]Model includes the exposure of interest and treatment groups.

[b]Model includes the exposure of interest, treatment groups, length of time on dialysis and current ACEI/ARB use.

[c]Model did not allow inclusion of any covariate.

[d]Model includes the exposure of interest, treatment groups, study visits, length of time on dialysis, and current ACEI/ARB use.

[e]Model includes the exposure of interest, treatment groups and study visits.

[f]Model includes the exposure of interest.
